# Factors associated with suicide attempts in the antecedent illness trajectory of bipolar disorder and schizophrenia

**DOI:** 10.1186/s40345-023-00318-3

**Published:** 2023-12-08

**Authors:** Alessandro Miola, Manuel Gardea-Reséndez, Javier Ortiz-Orendain, Nicolas A. Nunez, Mete Ercis, Brandon J. Coombes, Manuel Fuentes Salgado, Peggy M. Gruhlke, Ian Michel, J. Michael Bostwick, Alastair J. McKean, Aysegul Ozerdem, Mark A. Frye

**Affiliations:** 1 Department of Psychiatry & Psychology, Mayo Clinic, Rochester, MN USA; 2 Department of Neuroscience (DNS), University of Padova, Padua, Italy; 3 Department of Psychiatry, Universidad Autónoma de Nuevo León, Monterrey, México

**Keywords:** First episode mania, First episode psychosis, Psychiatric antecedents, Suicidality, Risk factors, Early intervention

## Abstract

**Background:**

Factors associated with suicide attempts during the antecedent illness trajectory of bipolar disorder (BD) and schizophrenia (SZ) are poorly understood.

**Methods:**

Utilizing the Rochester Epidemiology Project, individuals born after 1985 in Olmsted County, MN, presented with first episode mania (FEM) or psychosis (FEP), subsequently diagnosed with BD or SZ were identified. Patient demographics, suicidal ideation with plan, self-harm, suicide attempts, psychiatric hospitalizations, substance use, and childhood adversities were quantified using the electronic health record. Analyses pooled BD and SZ groups with a *transdiagnostic approach* given the two diseases were not yet differentiated. Factors associated with suicide attempts were examined using bivariate methods and multivariable logistic regression modeling.

**Results:**

A total of 205 individuals with FEM or FEP (BD = 74, SZ = 131) were included. Suicide attempts were identified in 39 (19%) patients. Those with suicide attempts during antecedent illness trajectory were more likely to be female, victims of domestic violence or bullying behavior, and have higher rates of psychiatric hospitalizations, suicidal ideation with plan and/or self-harm, as well as alcohol, drug, and nicotine use before FEM/FEP onset. Based on multivariable logistic regression, three factors remained independently associated with suicidal attempts: psychiatric hospitalization (OR = 5.84, 95% CI 2.09–16.33, *p* < 0.001), self-harm (OR = 3.46, 95% CI 1.29–9.30, *p* = 0.014), and nicotine use (OR = 3.02, 95% CI 1.17–7.76, *p* = 0.022).

**Conclusion:**

Suicidal attempts were prevalent during the antecedents of BD and SZ and were associated with several risk factors before FEM/FEP. Their clinical recognition could contribute to improve early prediction and prevention of suicide during the antecedent illness trajectory of BD and SZ.

## Introduction

Suicide is a multifaceted global public health concern that leads to more than 700,000 deaths/year [WHO 2021]. Psychological autopsy studies in high-income countries revealed that at least 90% of people who die of suicide had a mental disorder at the time of death [Cavanagh et al. 2003; Hawton and van Heeringen 2009; Phillips 2010]. Additionally, two-thirds of persons who died from suicide had mental health care contacts, both primary and specialty outpatient care, the year before death [Schaffer et al. 2016]. However, risk prediction of suicide is challenging, owing to the low base rate of suicide, weakness of existing suicide prediction tools and their low predictive validity, especially for suicide mortality, and lack of evidence that targeted clinical interventions are effective in preventing suicide completion [Goldney 2000; Belsher et al. 2019; Kessler et al. 2020]. In this context, a prior history of suicide attempt is considered one of the most robust predictors of an eventual completed suicide [Gibb et al. 2005; Christiansen and Jensen 2007]. A previous observational cohort study using the Rochester Epidemiology Project (N = 1490 suicide attempters) supported that suicide attempts had a greater lethal risk than previously thought with approximately 60% of individuals who completed suicide died on their index attempts, and with more than 80% of subsequent completed suicides occurring within a year of the initial attempt [Bostwick et al. 2016].

Among subjects with severe mental illness, individuals with bipolar disorder (BD) and schizophrenia (SZ) have higher rates of suicidal behavior when compared to the general population. Specifically, the estimated pooled lifetime prevalence of suicide attempts was 26.8% (95% CI 22.1–31.9%) for individuals with SZ [Lu et al. 2019] and 33.9% (95% CI 31.3–36.6%) for those with BD [Dong et al. 2019]. Moreover, suicidality has been reported as a prevalent dimension in the prodromal phase of BD [Correll et al. 2007, 2014], SZ [Andriopoulos et al. 2011], and in those at clinical high-risk for psychosis [Haining et al. 2021]. Although the phenotypical overlap between the psychiatric antecedents in these disorders calls for a simultaneous investigation into both illness trajectories [Léger et al. 2022]. For example, only a few studies have analyzed the degree of suicidal behavior between patients with BD and SZ prior to syndromal expression of BD and SZ [Kafali et al. 2019; Léger et al. 2022; Verdolini et al. 2022] without specifically investigating factors associated with suicide attempts. However, it is crucial to prioritize future research efforts in identifying populations at risk for initial suicidal attempts due to the significant lethality associated with them [Bostwick et al. 2016]. To the best of our knowledge, no previous study used a transdiagnostic approach for investigating suicidality in the antecedent illness trajectory of BD and SZ.

In our previous community-based epidemiological cohort study, we reported similar duration of psychiatric antecedents for BD (7.39 ± 6 years) and SZ (8.2 ± 6 years) suggesting similar but non-identical illness trajectories before the first episode mania (FEM) and first episode psychosis (FEP) [Ortiz-Orendain et al. 2023], as it has been previously suggested [Correll et al. 2007; Olvet et al. 2010]. Indeed, the few studies that have compared the antecedents of SZ and BD have found a significant overlap in the illness trajectory prior to FEM/FEP, revealing striking resemblances between these two conditions in clinical features, including symptom profile, premorbid adjustment, prescribed medications, substances used, and historical diagnoses [Correll et al. 2007; Olvet et al. 2010; Rietdijk et al. 2011; Chan et al. 2019; Kafali et al. 2019; Verdolini et al. 2022], with some authors suggesting that the antecedents between these two disorders are “indistinguishable” [Olvet et al. 2010]. Furthermore, a growing body of literature suggests that BD and SZ share commonalities across multiple domains, including genetic risk [Lichtenstein et al. 2009; Mullins et al. 2021], neuroimaging correlates [Dobri et al. 2022; Rootes-Murdy et al. 2022; Koen et al. 2023], and cognitive profiles [Pradhan et al. 2008; Olvet et al. 2010; Jiménez-López et al. 2017]. Such evidence supports that SZ and BD lie along a spectrum that argues for a dimensional approach to inform preventive strategies, particularly in the illness trajectory prior to full-blown diagnostic presentation of these disorders.

In this study, we aimed to investigate the prevalence and factors associated with suicide attempts during the illness trajectory between the onset of first symptoms and the full development of a FEM or FEP.

## Methods

### Search strategy

All residents from Olmsted County, MN USA born after 1985 and diagnosed with BD or SZ were identified using the Rochester Epidemiology project (REP). The REP is a population-based registry that allows to retrospectively analyze the history of individuals who developed an illness while mitigating risks of sampling, recall, referral, and response biases [Allebeck et al. 2009; Maret-Ouda et al. 2017]. The REP contains sociodemographic and clinical information of all residents of Olmsted County who had received treatment from 1986 onwards and has shown its validity in previous studies both for BD and SZ [Capasso et al. 2008; Prieto et al. 2016; Gardea-Resendez et al. 2023; Ortiz-Orendain et al. 2023].

A detailed description of the search strategy and case ascertainment has been extensively described in a previous report conducted by our group [Ortiz-Orendain et al. 2023]. After conducting an initial screening phase that involved utilizing diagnostic codes and systematically evaluating the health records of 1335 patients for DSM-5 criteria of BD and SZ as determined by two psychiatrists (authors JOO or MGR), we proceeded to search for FEM or FEP as described by Breitborde et al. (2009) [Breitborde et al. 2009]. Any disagreements regarding case ascertainment were resolved by consulting a panel consisting of three senior psychiatrists (AMc, AO, and MAF).

### Population

Once we identified the SZ and BD cases with FEP or FEM, patients’ demographics, first-episode data, and psychiatric history prior to FEM/FEP were extracted using a standardized version to collect data created with RedCap [Harris et al. 2019]. Codes for mental health disorders were classified according to the spectrums of DSM-5 [APA 2013]. Specifically, we collected data for the following variables: suicidal ideation with plan, self-harm, suicide attempts, psychiatric hospitalizations, alcohol and substance use, previous childhood adversities, and psychiatric diagnosis during the illness trajectory before FEM/FEP. A full description of the other extracted variables has been thoroughly documented in recent reports carried out by our group [Gardea-Resendez et al. 2023; Ortiz-Orendain et al. 2023]. Considering the lack of consensus on the operationalization of the prodrome phase [Geoffroy et al. 2017; Kupka et al. 2021], the psychiatric antecedent onset was defined as the date in which the first psychiatric symptoms, including neurodevelopmental disorders, was first coded in the patients’ health record or the first time a mental health problem was reported in the problem list of a clinical note [Ortiz-Orendain et al. 2023]. The first episode was defined as the clinical encounter during which an individual was first noted to exhibit symptoms of psychosis or mania [Breitborde et al. 2009]. For this study, a suicide attempt was defined as a self-injurious behavior with the intention to die, whereas self-harm with no suicidal intention was reported as a separate outcome, as it has been previously done in other investigations [Verdolini et al. 2022].

The current retrospective population-based study was approved by the institutional review boards (IRBs) of Mayo Clinic (IRB # 20–006720 and 20–008773) and Olmsted Medical Center (Approval dates: 11/20/2020 and 04/22/2021) who provided a Health Insurance Portability and Accountability Act (HIPAA) waiver, in line with state, federal, and international recommendations. Hence, written informed consent was not required for passive medical record review in the REP [Rocca et al. 2018].

### Statistical analysis

Prodromal analyses pooled BD and SZ groups, given the two diseases were not yet differentiated prior to the full development of a FEM or FEP and the evidence of phenotypical overlap between the antecedent illness trajectory in these disorders [Correll et al. 2007; Olvet et al. 2010; Léger et al. 2022]. Sociodemographic and selected clinical data associated with suicide attempts were compared between subjects with versus without at least one suicide attempt using chi-square tests (χ^2^) for categorical measures and analysis of variance (two-sample t-tests) for continuous measures. Due to the high correlation between the aforementioned variables, we used a multivariate logistic regression model to identify factors that were independently associated with suicide attempts. However, because of the limited sample size of the study, we could not include all variables into one model. Instead, we tested several multivariate logistic regression models using a forward–backward stepwise approach for variable selection. Given the known strong relationship between certain variables such as suicide attempts and psychiatric hospitalizations and self-harming behavior [Verdoux et al. 2001; Goldstein et al. 2005; Robinson et al. 2010; Beckman et al. 2016; Olfson et al. 2016], we also repeated this stepwise approach while omitting these variables from the selection procedure. Model performance was then compared and the two models (with and without previous hospitalization, self-harm and ideation included) with the lowest Akaike Information Criterion (AIC)—which favors more parsimonious models—were selected [Hosmer et al. 2013]. Odds ratios (OR) with confidence intervals (CI) were used as a measure of effect size. Statistical analyses were performed using Jamovi (https://www.jamovi.org/).

## Results

### Participants characteristics

This study included 205 patients who met diagnostic criteria for a DSM-5 SZ (N = 131) and BD (N = 74) with FEP or FEM. Of the 205-study sample, 28 (13.7%) experienced suicidal ideation with plan, 41 (20.0%) had a previous self-harm behavior, and 39 (19%) attempted suicide during the illness trajectory prior to FEM or FEP (Table 1).

**Table 1:** Demographic and suicidality (suicidal ideation with plan, self-harm, and suicide attempts) of 205 patients during psychiatric antecedents before FEP or FEM

Variables	Magnitude [95% CI]	
	BD	SZ
Subjects	74	131
Women, n (%)	29 (39.2)	25 (19.1)
Age at FEP/FEM, mean (SD)	21.3 (3.56)	20.4 (3.88)
Psychiatric hospitalizations, n (%)	20 (27.0)	24 (18.3)
Suicidal ideation with plan, n (%)	12 (16.2)	16 (12.2)
Self-harm, n (%)	18 (24.3)	23 (17.6)
Suicide attempt, n (%)	17 (23.0)	22 (16.8)

*FEP* First episode psychosis, *FEM* First episode mania, *BD* Bipolar Disorder, *SZ* Schizophrenia

Demographic and clinical characteristics of 205 patients with and without suicide attempts during the illness trajectory before FEM/FEP are compared in Table 2.

**Table 2:** Sociodemographic, clinical characteristics of 205 patients with and without suicide attempts during psychiatric antecedents before FEP or FEM

Variables	Attempted suicide (N = 39)	No suicide attempt (N = 166)	*t*-score or χ^2^	*p*-value
Women, n (%)	17 (43.6)	38 (22.9)	6.89	0.009
Diagnosis:				
- BD, n (%)	17 (43.6)	57 (34.3)		
- SZ, n (%)	22 (56.4)	109 (65.7)	1.17	0.279
Age at FEP/FEM, mean (SD)	21.7 (4.37)	20.5 (3.61)	2.18	0.146
Psychiatric hospitalization before FEP or FEM, n (%)	24 (61.5)	20 (12.0)	45.9	< 0.001
Psychiatric diagnosis before FEP or FEM:				
- ADHD, n (%)	18 (46.2)	55 (33.1)	2.34	0.126
- Autism spectrum disorder, n (%)	1 (2.6)	6 (3.6)	0.106	0.745
- Depressive disorder spectrum, n (%)	31 (79.5)	78 (47.0)	13.4	< 0.001
- Anxiety disorders, n (%)	22 (56.4)	51 (30.7)	9.09	0.003
- Adjustment disorders, n (%)	16 (41.0)	38 (22.9)	5.35	0.021
- Sleep disorders, n (%)	9 (23.1)	16 (9.6)	5.33	0.021
- PTSD, n (%)	6 (15.4)	11 (6.6)	3.19	0.074
- Eating disorder, n (%)	4 (10.3)	5 (3.0)	3.95	0.047
- Conduct disorder, n (%)	6 (15.4)	16 (9.6)	1.09	0.297
- Personality disorders, n (%)	6 (15.4)	10 (6.0)	3.85	0.05
Self-Harm before FEP or FEM, n (%)	21 (53.8)	20 (12.0)	34.5	< 0.001
Suicidal ideation with plan, n (%)	16 (41.0)	12 (7.2)	30.6	< 0.001
Alcohol use, n (%)	25 (64.1)	68 (41.0)	6.82	0.009
Methamphetamine/MDMA use, n (%)	12 (30.8)	21 (12.7)	7.68	0.006
Cannabis use, n (%)	31 (79.5)	107 (64.5)	3.24	0.072
Cocaine use, n (%)	7 (17.9)	12 (7.2)	4.32	0.038
Abuse Hypnotics/benzodiazepines/barbiturates, n (%)	8 (20.5)	10 (6.0)	8.28	0.004
Nicotine use, n (%)	25 (64.1)	53 (31.9)	13.9	< 0.001
Childhood adversities:				
- Sexual abuse, n (%)	7 (17.9)	22 (13.3)	0.57	0.449
- Physical abuse/neglect, n (%)	10 (25.6)	27 (16.3)	1.88	0.171
- Emotional abuse/neglect, n (%)	10 (25.6)	24 (14.5)	2.85	0.091
- Domestic violence, n (%)	16 (41.0)	32 (19.3)	8.33	0.004
- Bullying, n (%)	7 (17.9)	9 (5.4)	6.89	0.009

*FEP* First episode psychosis, *FEM* First episode mania, *BD* Bipolar Disorder, *SZ* Schizophrenia, *ADHD* Attention Deficit-Hyperactivity Disorder, *PTSD* Posttraumatic stress disorder, *MDMA* 3,4-methylenedioxy-methamphetamine

Those with suicide attempt did not significantly differ in terms of diagnosis (BD = 22.97% and SZ = 16.79%, respectively, p = 0.279) nor the age of first episode when compared with those who did not experience any suicide attempt (21.7 vs. 20.5 years, p = 0.146). When compared with patients who did not attempt suicide, those with suicide attempts were more likely to be women (22.9 vs. 43.6%, respectively, p = 0.009) and had higher rates of psychiatric hospitalizations during the illness trajectory before FEM/FEP (12 vs. 61.5%, p < 0.001). As expected, those that attempted suicide were more likely to experience suicidal ideation with plan (41 vs. 7.2%, p < 0.001) and exhibited higher rates of self-harm during the illness trajectory before FEP/FEM when compared to those who did not (53.8 vs. 12%, p < 0.001). Additionally, those with suicide attempts were more likely to have had witnessed domestic violence (41 vs. 19.3%, p = 0.004) and be victims of bullying behavior (17.9 vs. 5.4%, p = 0.009). Patients attempting suicide during the illness trajectory before FEM/FEP displayed higher rates of depressive disorders (79.5 vs. 47%, p < 0.001), anxiety disorders (56.4 vs. 30.7%, p = 0.003), and adjustment disorders (41 vs. 22.9%, p = 0.021), along with sleep disorders (23.1 vs. 9.6%, p = 0.021), disordered eating behaviors (10.3 vs. 3%, p = 0.047) and personality disorders (15.4 vs. 6%, p = 0.05) when compared to those who did not. Lastly, those with suicide attempts were more likely to use nicotine (64.1 vs. 31.9%, p < 0.001), alcohol (64.1 vs. 41%, p = 0.009), methamphetamines (30.8 vs. 12.7%, p = 0.006), cocaine (17.9 vs. 7.2%, p = 0.038), and to abuse hypnotics, benzodiazepines, and barbiturates (20.5 vs. 6%, p = 0.004) in comparison to those who did not attempt suicide before FEM/FEP.

### Multivariable modeling

We first ran our stepwise approach using all of the variables included in Table 2. The best model included three factors which were all independently associated with suicidal attempts during the illness trajectory before FEM/FEP: [a] psychiatric hospitalizations (OR = 5.84, 95% CI 2.09–16.33, p < 0.001), [b] self-harming behavior (OR = 3.46, 95% CI 1.29–9.30, p = 0.014), [c] nicotine use (OR = 3.02, 95% CI 1.17–7.76, p = 0.022) (Table 3). The variance explained by the model was 39% (Nagelkerke R-Squared = 0.390, AIC = 135).

**Table 3:** Logistic regression model selected using stepwise approach to identify factors independently associated with suicide attempts

Factor	OR (95% CI)	*p-value*
Psychiatric hospitalization	5.84 (2.09–16.33)	< 0.001
Self-harm	3.46 (1.29–9.30)	0.014
Nicotine use	3.02 (1.17–7.76)	0.022
Female sex	2.33 (0.83–6.56)	0.109

Because factors such as previous hospitalization, self-harming behavior, and suicidal ideation are well-known risk factors for future attempts [Beckman et al. 2016; Olfson et al. 2016], we also investigated a model which excluded these factors to determine if other variables could also be associated with suicide attempt during the illness trajectory before FEM/FEP. In this second stepwise procedure, the best model retained four factors all of which were also independently associated with suicidal attempts: [a] female sex (OR = 3.09, 95% CI 1.29–7.39, p = 0.011), [b] depressive disorder diagnosis (OR = 2.71, 95% CI 1.10–6.69, p = 0.030), [c] nicotine use (OR = 4.78, 95% CI 1.98–11.54, p < 0.001), [d] bullying (OR = 5.09, 95% CI 1.44–18.00, p = 0.012) (Table 4). This model did not perform as well as the model using known factors for suicide attempts but still explained 28% of the variance (Nagelkerke R-Squared = 0.283, AIC = 172).

**Table 4:** Logistic regression model using stepwise approach after removing psychiatric hospitalization and self-harming behavior

Factor	OR (95% CI)	*p-value*
Female sex	3.09 (1.29–7.39)	0.011
Depressive disorder spectrum	2.71 (1.10–6.69)	0.030
Nicotine use	4.78 (1.98–11.54)	< 0.001
Bullying	5.09 (1.44–18.00)	0.012
Domestic violence	1.53 (0.65–3.59)	0.325

## Discussion

This community-based epidemiological cohort study which included 205 subjects who met DSM-5 diagnostic criteria for SZ (N = 131) and BD (N = 74) aimed to investigate transdiagnostically the frequency of suicide attempts as well as to identify sociodemographic and clinical features associated with suicide attempts during the illness trajectory prior to a FEM/FEP.

Our findings revealed that one fifth (N = 39, 19%) of patients attempted suicide during the illness trajectory before FEM/FEP, while suicidal ideation with plan was found in 13.7% (N = 28) and self-harming behaviors in 20% (N = 41) of the patients studied. There were no significant differences in suicidality between the two diagnoses (BD vs. SZ), which is in accordance with recent reports [Lèger et al. 2022; Verdolini et al. 2022].

Although a broad range of factors were associated with suicide attempts before FEM/FEP in the univariate logistic regression models (Table 2), female sex, psychiatric hospitalizations, self-harming behavior, depressive disorders, nicotine use, and being the victims of bully behavior were found to be independently associated with suicide attempts in the multivariable models. Our work is in accordance with earlier research that examined suicide risk factors in major affective disorders (MDD and BD) identifying a greater number of hospitalizations for depression and early abuse and trauma [Baldessarini et al. 2019; Miola et al. 2023]. In addition, large epidemiological studies in the US have reported that bullying behavior was associated with an increase frequency of suicide ideation, plan and attempts in children [Kim et al. 2008] which emphasizes the need to carefully monitor, and tailor targeted early interventions in this population. As expected, previous or current depressive symptoms have been associated with suicide attempts both in patients with FEM [Khalsa et al. 2008] and FEP [Barrett et al. 2010; Andriopoulos et al. 2011; Sanchez-Gistau et al. 2013]. Moreover, our findings of higher rates of psychiatric hospitalization and non-suicidal self-injurious behavior in those who attempted suicide are consistent with previous reports which focused on youth with BD [Goldstein et al. 2005, 2012] and FEP [Verdoux et al. 2001; Robinson et al. 2010].

There has been ample literature that has shown an association between substance use and higher prevalence of suicide attempts in affective disorders [Tondo et al. 1999; Miola et al. 2023] and SZ [Fuller-Thomson and Hollister 2016]. Interestingly, our study has shown a higher association with nicotine, alcohol, meta-amphetamines, cocaine and abuse of hypnotics, benzodiazepines, and barbiturates in the illness trajectory before FEM/FEP. The high prevalence and bidirectional association between suicide attempts and substance use has also been suggested in previous studies. For example, a Swedish population study of 13,352 men and 5542 women examined death caused by suicide and highlighted that alcohol, cannabis, and central stimulants were associated with higher violent methods of suicide [Lundholm et al. 2014]; all these underscoring the need to carefully review patients use or abuse of substances as well as accessibility to weapons. Similarly, in a Danish register-based cohort study analyzing data from people diagnosed with SZ (n = 35,625), BD (n = 9279), depression (n = 72,530), or personality disorder (n = 63,958), having any substance use disorder was associated with at least a threefold increased risk of completed suicide compared with those without substance use disorder [Østergaard et al. 2017]. Of note, our findings underscoring the association of nicotine use and suicide attempts during psychiatric antecedents before FEM/FEP (significant for in both multivariable logistic regression models), even after controlling for well-known confounding factors including female sex, previous childhood adversities, depressive disorders, and co-occurring alcohol and substance use [Tomori et al. 2001], is in accordance with a study by Andriopoulus and colleagues [Andriopoulus et al. 2011]. As also seen in the current study, the latter report emphasized that tobacco smoking and depressive mood were associated with prodromal suicide attempts in patients with SZ [Andriopoulus et al. 2011]. Moreover, tobacco smoking has been reported as a potentially independent risk factor for suicide attempts in BD [Ostacher et al. 2009; Ducasse et al. 2015]. Interestingly, tobacco smoking, both alone and comorbid with alcohol use disorders, depressive polarity of BD onset, and female sex were independently associated with recurrent suicide attempts in a cohort of 916 patients with BD [Icick et al. 2019].

However, it is important to acknowledge certain limitations of the present study. First, the retrospective study design may introduce inherent limitations such as information bias and missing data, which may limit our ability to establish causality in the current findings. Nonetheless, previous REP studies have demonstrated validity and reliability, as evidenced by previous reports [Capasso et al. 2008; Prieto et al. 2016; Gardea-Resendez et al. 2023; Ortiz-Orendain et al. 2023]. Second, the REP demographics may limit the study’s generalizability due to a less diverse population (higher prevalence of white population), and of higher socioeconomic status [St Sauver et al. 2012]. Third, suicidal ideation was evaluated by clinicians without the use of standardized scales within the real-world clinical practice setting and was extracted from clinical notes of patients’ health records, which may have led to potential underreporting of such cases. Fourth, recognition of suicide attempts is more difficult than suicide because nonviolent attempts may not be reported, can easily be concealed, and may be considered accidents or nonvoluntary acts. As a result, reported rates of suicide attempts are at risk of misdiagnosis and underreporting [Bachmann 2018]. Nevertheless, the REP represents a unique database in the United States that permits population-based research with medical, and continuity of care data collected by healthcare professionals [St Sauver et al. 2012].

On the other hand, our study has some strengths such as the thorough case ascertainment by psychiatrists and the quality of the extracted data from clinical notes which can minimize the possibility of a recall bias.

In conclusion, our study brings to the forefront the need to further examine and carefully monitor for clinical risk factors during the illness trajectory before FEM/FEP. Larger studies with prospective longitudinal design may be needed to validate some of these findings as well as to quantify risk for suicidality in this vulnerable population as to tailor effective preventive measures.

## Data Availability

The data that support the findings of this study are available on request from the corresponding author. The data are not publicly available due to privacy or ethical restrictions.
